# Notes and News

**Published:** 1888-11-03

**Authors:** 


### Notes and News.

Collected and Communicated.

The Committee of the Falkirk Cottage Hospital Fund have purchased Salton Cottage.

The Leeds Workpeople's Hospital Fund amounts to over fifty pounds.

Hospital Sunday at Devonport was observed by the Catholics, but, with few exceptions, not by the other Dissenters.

The Wycombe Board of Guardians have increased their subscription to the Cottage Hospital, because they find it of the greatest benefit to the town.

The Royal Commission on the Blind, the Deaf, and the Dumb have just concluded a tour of inspection, during which they have visited Lanark, Glasgow, Oldham, etc.

A distraint sale for non-compliance with the Vaccination Act took place last week at the instance of the Andover Board of Guardians. The proceedings were of the liveliest description.

The Cheltenham Town Council have requested the Delancey Hospital to take typhoid cases, as the General Hospital has lately limited the number of beds allotted to such cases. The request was kindly complied with, but only as a temporary arrangement.

Monkwearmouth Dispensary is sadly in need of a surgical ward, but also is sadly in want of funds. Chancellor Sanderson has, however, found a cheerful way of raising money, and has inaugurated a series of concerts to be carried on during the winter months, which will entertain the healthy and benefit the suffering.

Now we are going to have a real test of Dr. Gamalia's method of inoculating for cholera. He proposed to the French Academy that he should experiment with it upon himself, and after long hesitation his proposal has been accepted. We trust that the plucky doctor will come well out of the experiment.

The Hull Orthopædic Hospital, which was for many years located over a chemist's shop, has at last secured more suitable premises. Massage and electro-massage are carried on by Mr. Webster and Mrs. Shaw, with considerable success. Last week the Chief Constable of Hull and a few gentlemen visited the hospital, and inspected the casts and photographs which are kept, of the worst cases.

Last Sunday was Hospital Sunday at Worcester, and collections were made in most of the churches and chapels in the city and county, on behalf of the infirmary, which admits over 1,000 in-patients yearly; the dispensary, which treats 8,000 patients yearly; and the Ophthalmic Hospital, which treats about 100 in and 700 out-patients in the year. The collections are expected to amount to over £500.

Richmond has for the first time indulged in a Hospital Saturday, and was luckily blessed with a fine day. The Thames watermen, who take a great interest in this well-managed little hospital, lent boats decorated with flags, which were placed at prominent street corners. The procession consisted chiefly of bands, banners, and money-boxes, and made a great deal of noise and collected £140.

Mr. Otto Jackson Kauffmann, M.B. London, M.R.C.S., has been appointed house physician to the Seamen's Hospital, vice Dr. Trevelyan, resigned; Mr. Walter Fowler, M.A., M.B., B.A. Cantab, B.Sc. London, F.R.C.S. Eng., has been appointed surgeon to the Well Street Branch of the Seamen's Hospital, vice Mr. Cotes, resigned; and Mr. W. P. Want, M.P.S., has been appointed dispenser at Well Street Branch of the Seamen's Hospital, in the place of the late Mr. Leadbeater.

The City Police Orphanage, Strawberry Hill, has received £250 from the executors and trustees of the will of the late Miss Louisa McKellar, of Argyll Lodge, Clapham Park. The Church of England Central Society for providing Homes for Waifs and Strays has received £200, bequeathed by the same lady, and the sum of £100 was also sent to the East London Nursing Society from the same source. The Fishmongers' Company have given a donation of £250 to the funds of the National Sea Fisheries Protection Association.

Prince Albert Victor laid the foundation-stone of a new wing to the Ancoats Hospital during his visit to Manchester. The Prince first inspected the wards, and was then presented with an address by the honorary secretary, Mr. Forrest. In the lower parts of Ancoats, and in Mill Street, where the Prince saw the thousands of workers of Manchester, the manifestations of loyalty were particularly hearty. His presence at such a function was evidently appreciated.

Mr. Joseph Pearson has entered into residence at the Borough Hospital, Sheffield, as medical superintendent. The vacancy was caused by the resignation of Dr. Willey, who is going in for a private practice, much to the regret of all the inmates of the hospital. Mr. Pearson's former appointment was at Lodge Moor, the hospital which was built at the cost of £13,000 to meet the requirements of the recent severe epidemic of small-pox. Mr. Percy Edwards has succeeded him there, but at present has no patients.

At the Poor Law Conference at Chester, on October 19th, Mr. Mellor, of Oldham, read a paper on "Hospitals for Infectious Diseases, and the Notification of Disease." The paper consisted largely of correspondence between the Oldham Board of Guardians and the Corporation of Middleton, upon this question. After much discussion, it was finally resolved: "That this Conference is strongly of opinion that the provision of hospitals, or other suitable accommodation for infectious disease by sanitary authorities should be made compulsory, and that the notification of such disease should be made compulsory also."

At a late meeting of the Cork Dispensary Committee, Dr. Golding attended to explain a sad case. A respectable man, working as a slater was suffering from blood-spitting, and Dr. Golding attended him on a red ticket. He ordered his removal to the Union Hospital, and whatever time he got there he dropped dead on admission. Practically speaking, a ticket is issued by the relieving officer or guardian in the morning, and is never attended to until late at night. Dr. Golding said it was not for the purpose of casting reflection on anybody that he brought this matter forward; it was simply to call attention to the inefficiency of the poor-law system. Anybody had a right to come to him armed with a red ticket, but if he treated him he could not obtain relief for his family.

THE following vacancies are announced this week, the amount of salary in each case being given after the name of the institution :—

**Resident Medical Officers.**

St. Mark's Hospital for Fistula. Assistant Surgeon.
National Lying-in Hospital, Dublin. Assistant to the Master.
Wilts County Asylum. Assistant Medical Officer.—£100.
Chelsea Hospital for Women. Three Clinical Assistants.
Leeds Friendly Societies' Medical Association. Surgeon.—£200, residence, etc.
Female Lock Hospital, Harrow Road. Assistant House Surgeon. —Board, etc.
Medical Institution of Ancient Order of Shepherds, Bristol, Medical Officer.—£140, and unfurnished residence.

**Non-resident Medical Officers.**

City of London Hospital for Diseases of the Chest. Assistant Physician
Wirral Hospital and Dispensary for Children. Honorary Medical Officer.
St. Mark's Hospital for Fistula. Honorary Surgeon.

THE Lord Mayor has presented the certificates and medallions of the St. John's Ambulance Association gained during the year by members of the City of London Police Force and officers and non-commissioned officers of the City Militia. The Lord Mayor thought that to no class of the community was knowledge of that kind more practically useful than to members of the police force. He was glad to see that so many of them were qualifying themselves to be of use in those sudden emergencies and accidents in the streets which were of every-day occurrence.

THE last report of the Anting Missionary Hospital in Pekin contains some interesting information in regard to certain classes of Chinese patients. Suicides are very common in Pekin, a strong extract of opium being most commonly employed for the purpose, but stabbing with a knife in the abdomen is common. In one case of this kind which was treated at the hospital the reason assigned for the act was that the man had applied to a friend for a loan of money and was refused. In order to spite the niggard he committed suicide that his spirit might come back and perpetually annoy the latter.

AT the annual meeting of the Society of Medical Officers of Health, held last week at the Holborn Restaurant (Mr. Alfred Hill, M.D., presiding), the secretary's report showed that by the amalgamation of the society with the North-Western Association, the Yorkshire Association, and the Birmingham and Midland Association, the number of members amounted to 369, including sixty-four associates. The total payments amounted to £197 18*s.* 1*d.*, leaving a balance of £42 3*s.* 11*d.* Dr. W. H. Corfield, M.D., M.A., F.R.C.P. (the President-elect), delivered an interesting address, entitled "The History of Sewage Disposal." After the meeting the members of the society dined together.

THE *Pall Mall Gazette* says: "The *Lancet* has come to the rescue of cigarette makers and smokers. Having organised an Analytical Sanitary Commission, it now publishes their report, which should be worth a good deal to the Egyptian cigarette makers, who were especially attacked by "Medicus" not long ago. Five well-known brands have been examined, and they are all reported to be made of genuine tobacco, free from foreign substances, opium, or "unclassified alkaloid." Still we are told that it is "probably safer" to avoid (how?) Salouk tobacco (which seems to mean Smyrna tobacco), lest the throat may be affected by its use. All which leaves us much as we were before as to our knowledge of the risk run by cigarette smokers. It would be interesting to know how it is that the generally terrorist *Lancet* is now rather well-disposed than otherwise to the cigarette, which, only a few years ago, it damned so heartily that *Punch* burlesqued its diatribe by a cleverly drawn skeleton of the cigarette-smoking 'masher.'"

THE new wing erected at the Enfield Cottage Hospital, Chase Side, in commemoration of the Jubilee year of Her Majesty's reign, and to be known as the "Victoria Wing," has been opened in the presence of a representative gathering, and amid many manifestations of popular approval. The new wing, which has been erected by Mr. John Brooks, at a cost of between £300 and £400, stands at the rear of the old structure, and has a northern aspect. The additional apartment is commodious, lofty, and well lighted, and will contain three beds. Thus the total number of beds that the institution will provide for patients will be increased to ten. It is the intention of the committee to devote the new addition principally to women and children.

MR. JOHN GRIFFITHS has bequeathed £4,000 each to the London Hospital, to Guy's, to St. Mary's, and to the Middlesex. Guy's has also got £2,000 from the trustees of the late Miss McKellar. The will of Mr. William Adam, late of Tenterden Street, Hanover Square, who died on July 27th last, was proved on October 11th. The testator bequeaths £500 each to St. George's Hospital (Hyde Park Corner), King's College Hospital, the Middlesex Hospital, St. Mary's Hospital (Paddington), and Charing Cross Hospital; £600 to the Hospital for Incurables (Putney); £400 to the Association for the General Welfare of the Blind; £300 each to the Hospital for Epilepsy and Paralysis (Regent's Park), St. Thomas's Hospital, the Hospital for the Diseases of the Throat (Golden Square), the Infirmary at Forfar, and the Cancer Hospital, Brompton.

THE Rev. H. W. Webb-Peploe, of St. Paul's, Onslow Square, preached two sermons on Sunday last on behalf of the Consumption Hospital, Brompton. He impressed upon his hearers that the hospital was in their own parish, and it was their duty and privilege to liberally support it. There had been about 38,000 in-patients and upwards of 350,000 out-patients under treatment for periods of from a few weeks to several months. The letters which the congregation had placed in his hands were extremely valuable to the poor sufferers who so often applied to him, and he was glad of the opportunity they afforded of giving substantial consolation. Mr. Webb-Peploe urged upon his hearers the importance of annual subscriptions. Both Mr. Peploe and the afternoon preacher, the Rev. A. B. G. Lillingston, spoke in eloquent and feeling terms of the benefits conferred by the institution, for which the collections amounted to £103, including several annual subscriptions.

THE Mayor of Macclesfield (Thomas Crew, Esq.) has added another to his many benevolent acts by contributing fifty guineas to the endowment fund of the infirmary, and also providing a good feast and entertainment for the patients, nursing sisters, and other residents, instead of having the usual municipal banquet at the close of his year of office. On Monday last the patients, with the sanction of the medical staff, had a good dinner and tea, consisting of fish, turkey, and game, with delicious puddings, jellies, grapes, and other ripe fruit. In the afternoon Punch and Judy performed for the amusement of the children, and in the evening the convalescent patients of all ages were brought into the children's ward, and a musical entertainment was given by a local troupe of amateur Christy Minstrels. Several of the governors and members of the medical staff, with their wives and families, were present, and all the arrangements were carried out successfully, and were greatly enjoyed by those who were well enough to be present. Hearty votes of thanks were accorded to the Mayor and Mayoress, and the pleasures of the day will live long in the memories of all who partook of their hospitality. The Mayor has also entertained the inmates of the workhouse, industrial school, and the almshouses.

